# Low-Cost, High-Impact Teaching in Pharmacology: A Mixed-Methods Study Assessing the Effectiveness of a Ball-and-Balloon Activity on Student Engagement and Learning Outcomes

**DOI:** 10.7759/cureus.97529

**Published:** 2025-11-22

**Authors:** Divyashanthi Chellathambi Malathi, Niraimathi Manikam, Vijaykishan Bheemavarapu, Dinesh Kumar N, Akilesh Ramasamy

**Affiliations:** 1 Pharmacology, Jawaharlal Institute of Postgraduate Medical Education and Research, Puducherry, IND; 2 Pathology, Jawaharlal Institute of Postgraduate Medical Education and Research, Puducherry, IND; 3 Anatomy, Jawaharlal Institute of Postgraduate Medical Education and Research, Puducherry, IND; 4 Pediatrics, Jawaharlal Institute of Postgraduate Medical Education and Research, Puducherry, IND; 5 Dentistry, Jawaharlal Institute of Postgraduate Medical Education and Research, Puducherry, IND

**Keywords:** active learning, affordable methods, competency-based medical education, low-cost innovation, low resource setting, medical education research, pharmacology education, thematic analysis

## Abstract

Background

Pharmacology education often relies on didactic lectures, leading to challenges in maintaining student engagement and translating abstract drug mechanisms into a concrete understanding. This is particularly challenging in resource-constrained settings where access to high-fidelity simulations is limited. In alignment with competency-based medical education (CBME) principles, there is a pressing need for affordable, innovative, and highly impactful teaching-learning (T-L) methods that foster deep conceptual understanding and positive professional attitudes.

Methods

A pre- and post-intervention, mixed-methods study was conducted among second-year MBBS (Bachelor of Medicine and Bachelor of Surgery) students (n=50) in Puducherry, India. The topic of instruction was 'Drugs acting on the uterus.' The intervention involved a 60-minute didactic lecture followed immediately by a 15-minute hands-on Ball-and-Balloon activity, where students used a small plastic ball (representing the fetus) inside an inflated, inverted balloon (representing the uterus) to visualize the actions of uterotonic and tocolytic drugs. Knowledge was assessed using a validated 10-point questionnaire (pre- and post-test). Student feedback on engagement and attitude was collected via Likert scales and open-ended questions. Quantitative data were analysed using a paired t-test, and qualitative feedback underwent thematic analysis.

Results

The mean post-test score (*x*=9.2±0.8) was significantly higher than the mean pre-test score (*x*=7.0±1.5), demonstrating a substantial knowledge gain (*p*<0.001). Qualitative analysis identified seven major themes: Creative do-it-yourself learning, learning through positive psychology, reinforcing knowledge, concept-based learning, metacognition experience, positive inclination towards the subject, and multisensory learning experience. Students emphasized the activity's effectiveness in converting abstract concepts into a tangible, memorable experience.

Conclusion

The Ball-and-Balloon activity is a highly effective, low-cost T-L strategy that significantly improves student knowledge and fosters a positive attitude and deeper engagement with pharmacology. This model serves as a practical, replicable template for resource-limited institutions seeking to integrate active learning and visual teaching tools to meet contemporary CBME and professional development competencies

## Introduction

The undergraduate medical curriculum is undergoing a global transformation, shifting from traditional, knowledge-centric models to competency-based frameworks like CBME in India [1]. This transition necessitates the adoption of teaching-learning (T-L) methods that actively engage students, promote critical thinking, and integrate professional competencies (e.g., attitude, ethics, and communication (AETCOM)) alongside cognitive skills [2].

Pharmacology, the study of drug action, represents a significant challenge within the preclinical phase. It is inherently complex, involving abstract biochemical pathways, receptor interactions, and the subtle interplay of pharmacodynamics and pharmacokinetics. Traditional didactic lectures, which often rely heavily on rote memorization, frequently lead to low student engagement, a perception of the subject as 'dry' or 'boring,' and poor retention of conceptual knowledge [3,4].

The challenge is further compounded in resource-constrained settings, where institutions may lack the financial capacity to invest in high-fidelity simulations, expensive three-dimensional (3D) models, or sophisticated digital learning platforms. As noted by the World Health Organization, a significant proportion of medical schools in low- and middle-income countries still operate with limited access to essential digital and simulation tools [5]. This disparity necessitates a focus on low-cost, high-impact educational innovations that are both effective and universally scalable.

The present study addresses this critical gap by evaluating a simple, highly affordable, and hands-on activity, the Ball-and-Balloon model, designed to teach the mechanism of action of drugs acting on the uterus. This specific topic, involving the complex dynamics of uterine muscle tone, contraction, and relaxation, is particularly challenging to teach purely through text and diagrams. The study, conducted with prior ethics approval and grounded in the principles of educational research, aims to systematically assess the impact of this novel T-L method on: (i) Cognitive outcomes: measuring knowledge gain through pre- and post-intervention testing, and (ii) Affective outcomes: exploring changes in student engagement, attitude, and motivation towards pharmacology through detailed qualitative feedback. It is hypothesised that the introduction of this tangible, experiential model will result in statistically significant improvements in learning outcomes and a positive shift in student perception of the subject. The success of this low-cost innovation provides a replicable template for medical educators operating within resource-limited environments globally.

## Materials and methods

Study setting and design

The study was conducted in the Department of Pharmacology at a premier medical institution in India, the Jawaharlal Institute of Postgraduate Medical Education and Research (JIPMER), Karaikal, Puducherry, India. We employed a mixed-methods research design incorporating a pre- and post-intervention quantitative analysis (same-group design) and a qualitative thematic analysis of student feedback. The mixed-methods approach provided a robust understanding of the intervention, linking measurable knowledge gains with the students' experiential and affective responses [6]. Ethical approval for the study was obtained from the Institutional Ethics Committee (IEC) of JIPMER Karaikal (project number: JIP/IEC-0S/2024/628), prior to the commencement of the intervention.

Participants

The target population for the study comprised the entire cohort of second-year MBBS (Bachelor of Medicine and Bachelor of Surgery) students enrolled in the Pharmacology course. A total of 63 students who consented to participate and completed both pre- and post-tests were included in the final quantitative analysis. All participants voluntarily submitted feedback via Google Forms (Google LLC, Mountain View, California, United States) for the qualitative assessment.

Intervention: the Ball-and-Balloon activity

The topic was 'The mechanism of action and therapeutic uses of drugs acting on the uterus (uterotonics and tocolytics)'. The session was structured as a didactic lecture of 60 minutes followed by the Ball-and-Balloon activity of 15 minutes. The didactic lecture was a standard, interactive lecture covering the theoretical aspects of uterine physiology, different phases of the menstrual cycle, pregnancy, and the classification and mechanism of action of drugs like oxytocin, ergots, prostaglandins (uterotonics), and beta-2 agonists (tocolytics). Immediately following the lecture, students were engaged in the hands-on activity.

Description of the Ball-and-Balloon Model

**Figure 1:**
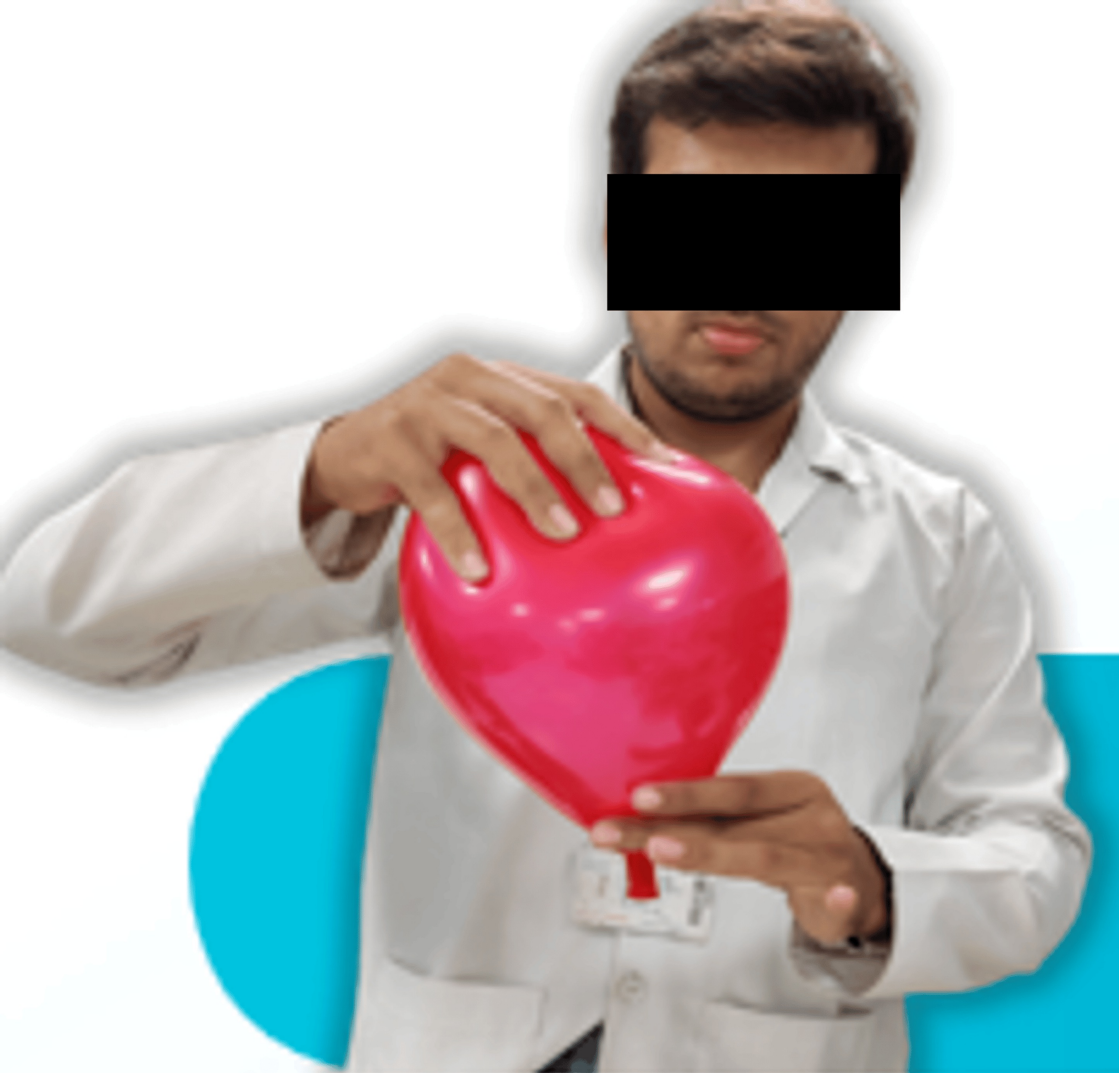
The Ball-and-Balloon activity

**Figure 2:**
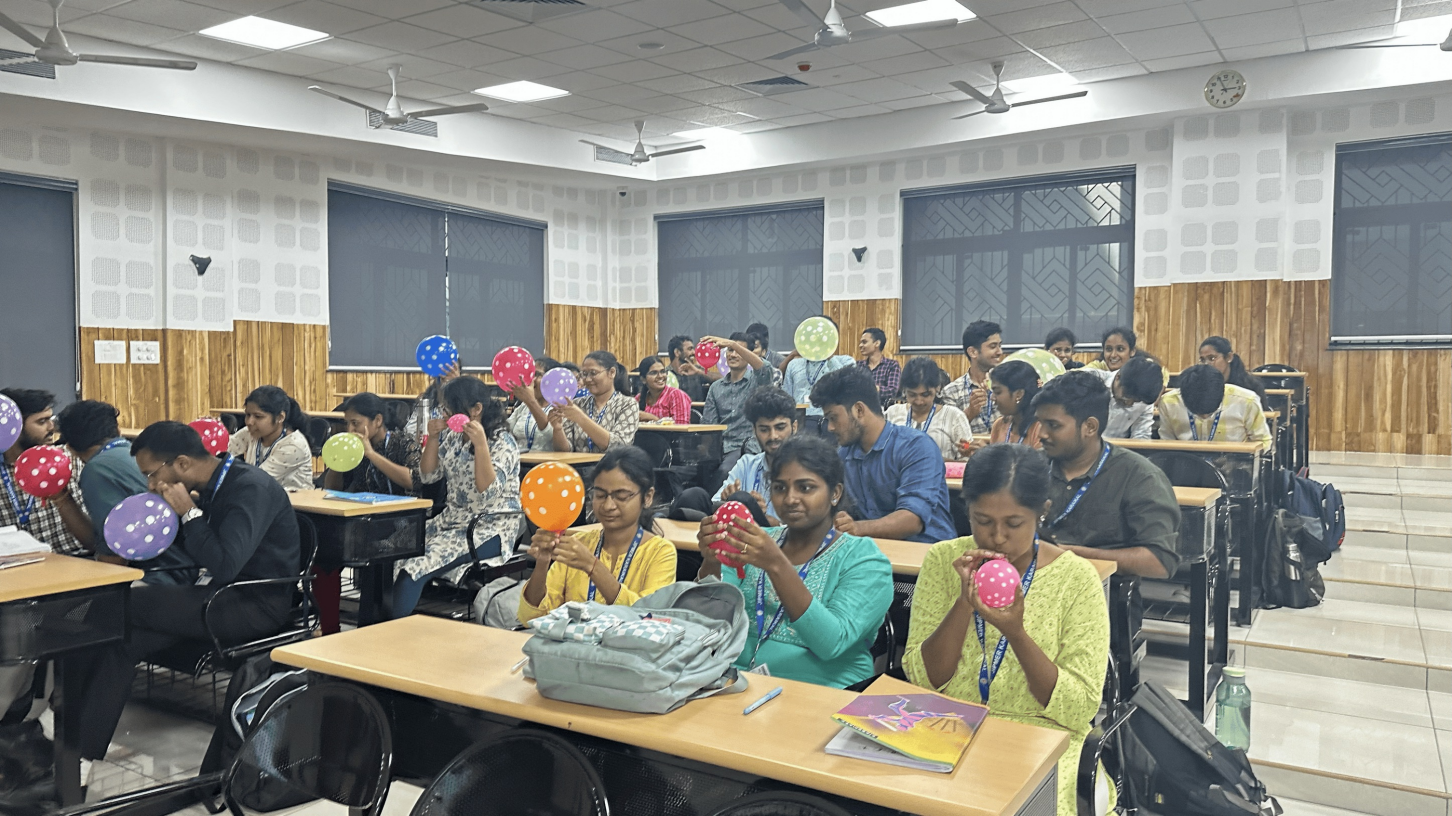
A classroom picture of students performing the activity Image Credit: Authors; consent was provided by the students for the use of photographs

Each student or pair of students received one standard party balloon (representing the uterus) and one small, lightweight plastic ball (representing the fetus) (Figures 1, 2). The plastic ball was carefully inserted inside the balloon, inflated, and inverted such that the ball snugly fits in the rim of the balloon.

Demonstration of Uterotonics (e.g., Oxytocin)

The instructor directed students to slowly release the air from the balloon's nozzle while simultaneously squeezing the exterior of the balloon gently (simulating the effect of the drug on the myometrium). This action causes the balloon wall to press down and expel the ball (fetus) forcefully and rapidly. The students visually and physically experienced the concept of increased uterine tone and powerful, coordinated contractions leading to expulsion.

Demonstration of Tocolytics (e.g., Beta-2 Agonists)

The instructor directed students to relax their grip on the balloon while simultaneously adding a small amount of air back into the balloon (simulating uterine relaxation). The balloon inflates slightly, the walls become less tense, and the ball (fetus) remains stable and unexpelled. This demonstrated the mechanism of reducing uterine tone to halt premature labor.

This activity served as an experiential learning tool, allowing students to bridge the gap between abstract textbook knowledge and its functional physiological representation.

Assessment tools

Knowledge Assessment (Quantitative)

A validated, 10-point, multiple-choice questionnaire (MCQ) was administered (see Appendices) as a pre-test immediately before the lecture and as a post-test immediately after the Ball-and-Balloon activity. The questionnaire included a mix of (i) Recall questions (lower-order cognition): Testing drug classification and primary mechanism, and (ii) Higher-Order Thinking Skills (HOTS) questions: Requiring application, analysis, and visualization, particularly related to clinical scenarios involving uterine tone changes (e.g., differentiating the mechanism of action of oxytocin versus a tocolytic drug in a case of delivery complications).

Scoring of Questions

The pre-test and post-test questionnaires were administered in printed form. Each correct response was awarded one mark. The answer keys were prepared and verified by subject experts from relevant departments. The scoring was carried out independently by two faculty members, and the data were entered into Microsoft Excel (Microsoft Corporation, Redmond, Washington, United States) for analysis.

Affective and Experiential Feedback (Qualitative)

Student feedback was collected anonymously using a structured Google Form. This form included: (i) Likert-scale items: Assessing perceived enjoyment, engagement, and conceptual clarity (not included in the final results tables for word count, but used to inform discussion), and (ii) Open-ended questions: Prompting students to describe their experience, what they liked most, how the activity impacted their understanding, and their overall attitude toward pharmacology.

Data analysis

Quantitative Data Analysis

Pre- and post-test scores were recorded and analysed using IBM SPSS Statistics for Windows, trial version 28 (IBM Corp., Armonk, New york, United States). As the data were paired (same students before and after intervention), a paired sample t-test was used to determine the statistical significance of the difference between the mean scores. A p-value of < 0.05 was considered statistically significant.

Qualitative Data Analysis

The open-ended responses were subjected to rigorous thematic analysis following the six-phase approach outlined by Braun and Clarke (2006) [7]: (i) Familiarizing the data: Reading and rereading the transcripts to achieve immersion, (ii) Generating initial codes: Identifying basic data segments related to the research aims (engagement, clarity, attitude), (iii) Searching for themes: Grouping codes into potential themes, (iv) Reviewing themes: Refining and collapsing themes to ensure they are coherent and distinct, (v) Defining and naming themes: Developing clear definitions and titles for the seven final themes, (vi) Producing the report: Linking the themes to the study narrative and presenting compelling, representative quotes.

## Results

Quantitative outcomes: knowledge gain

A total of 50 students completed both the pre-test and post-test. The analysis revealed a statistically significant improvement in knowledge scores following the intervention. The maximum achievable score was 10.

**Table 1:** Comparison of pre- and post-test knowledge scores (N=50)

Variable	Sample Size (n)	Mean Score (x)	Standard Deviation (SD)	Mean Difference (Post – Pre)	Paired (t)-test Value	(p)-value
Pre-test Score	50	7.0	1.5	2.2	11.64	< 0.001
Post-test Score	50	9.2	0.8			

The mean post-test score was 9.2 ± 0.8, showing a substantial increase of 2.2 points from the mean pre-test score of 7.0 ± 1.5. The highly significant p-value (< 0.001) confirms that the Ball-and-Balloon activity, in conjunction with the lecture, was highly effective in enhancing students' knowledge of the topic, particularly in applying higher-order thinking to drug actions.

Qualitative outcomes: student engagement and attitude

The thematic analysis of student feedback, following the Braun and Clarke methodology, identified seven distinct, recurring themes that captured the core impact of the intervention (Table 2).

**Table 2:** Summary of themes from qualitative thematic analysis of student feedback DIY: do-it-yoursef; PERMA: Positive Emotion, Engagement, Relationships, Meaning, and Accomplishment;

Theme No.	Theme Title	Definition	Representative Student Quote
1	Creative DIY learning	Recognition that such a highly effective educational tool could be implemented affordably, making it accessible to any institution regardless of budget.	"The innovative idea that such a high-impact learning experience could be delivered using such simple, low-cost, tangible materials is truly inspiring and shows creativity in teaching."
2	Learning through positive psychology (PERMA approach)	The activity’s novelty and hands-on nature served as a powerful antidote to the passive nature of traditional lectures, making the learning process enjoyable.	"It was very engaging and fun compared to the traditional PowerPoint style note-making format, which becomes boring after some time. The element of play made us instantly focus."
3	Positive Attitude towards Pharmacology	The intervention promoted a crucial positive shift in students’ affective domain, reducing the perception of the subject as being difficult or purely based on memorization.	"Before this, I thought Pharmacology was tough and boring due to endless rote memorization. This activity proved that even complex topics can be interesting and simplified using simple tools."
4	Knowledge reinforcement	Students reported a strong belief that the multi-sensory, experiential learning would aid in long-term memory consolidation and recall for their clinical practice.	"We applied everything we learned in the theory lecture using the simple ball and balloon. That concrete experience makes the concept easy for me to remember for my lifetime."
5	Metacognition experience	This method of T-L created an awareness of one's learning process and strategies.	"It covers the most important aspect of any lecture. The essence is simplified and it’s good for longer retention".
6	Concept based learning	This activity created a focus on understanding concepts rather than facts	“I liked it most when we could see the exact replica of the effect of the drug happening in front of my eyes”
7	Multisensory learning experience	Students felt that integration of visual and kinaesthetic senses led to immersive learning	"Seeing and doing practical improves interest and concentration."

## Discussion

The findings of this mixed-methods study strongly affirm the educational efficacy of the Ball-and-Balloon activity as a low-cost, high-impact teaching-learning tool in undergraduate Pharmacology. The results demonstrate significant improvements in both the cognitive and affective domains of learning, supporting the study’s hypothesis and validating the principles of experiential and active learning within medical education.

Cognitive impact and theoretical linkages

The quantitative results, showing a highly significant increase in post-test scores (p < 0.001), provide compelling evidence of effective knowledge transfer and, crucially, enhanced conceptual clarity. The topic of 'Drugs acting on the uterus' involves abstract concepts of smooth muscle tone and coordination, which are challenging to grasp from two-dimensional figures. By manipulating the balloon and ball, students engaged in experiential learning [8]. This hands-on interaction aligns with constructivist learning theory, where students actively construct knowledge and meaning from their experience rather than passively receiving information [9].

Furthermore, the significant gain in scores suggests that the model was particularly effective for higher-order thinking questions. When students physically simulate uterine contraction by squeezing the balloon, the mechanism of uterotonics like oxytocin is no longer a set of memorized words but a kinesthetic and visual reality, enabling them to apply this knowledge to clinical problem-solving. This tangible representation serves as a powerful anchoring concept for long-term memory retention and knowledge reinforcement (Theme 4).

Affective outcomes and the CBME framework

The qualitative data highlighted a crucial shift in the students’ affective domain, captured by Themes 2 and 3 (learning through positive psychology and a positive attitude). Medical educators, including Harden and Laidlaw, have long emphasized that effective learning must be FAIR (Focussed, Active, Individualized, and Relevant) and meet the CRISIS criteria (Convenience, Relevance, Individualization, Self-Assessment, Interest, and Systematic approach) [10]. The Ball-and-Balloon activity directly addresses the 'Interest' and 'Active' components, successfully breaking the 'monotony and boredom' associated with traditional lectures.

This positive change in attitude is perhaps the most significant long-term impact. When students perceive a subject as accessible, engaging, and relevant, their motivation increases, leading to deeper engagement and better learning outcomes across the entire curriculum [11]. The activity clearly imparts a sense of fun and novelty, contributing to a supportive and effective learning environment.

Educational significance: scalability and the resource gap

The most profound contribution of this study lies in its simplicity and affordability. In a global context where many institutions face financial constraints, the Ball-and-Balloon model demonstrates that effective, high-impact teaching need not be synonymous with high-tech simulation [5]. The materials required (a small ball and a balloon) are universally accessible and cost-effective, making the model instantly replicable and scalable across diverse resource settings. This principle of leveraging affordable, local resources for pedagogical innovation is vital for ensuring equitable access to quality medical education worldwide [12].

Limitations and future directions

While the study robustly supports the intervention's efficacy, it has limitations. The follow-up assessment (post-test) was immediate, meaning the results primarily reflect short-term memory gain. Future research should incorporate a delayed post-test (e.g., six months later) to definitively assess long-term retention. Furthermore, although the qualitative data provide strong evidence of an affective shift, future studies could employ validated, multi-item scales to quantitatively measure changes in attitude and motivation towards the subject.

The success of this model strongly suggests its applicability to other abstract topics in preclinical sciences, such as the mechanism of action of cardiac drugs (e.g., modeling cardiac output with a syringe and a container) or respiratory physiology. The Ball-and-Balloon activity thus serves as a template, encouraging educators in other disciplines to "Be FAIR" to their students by developing their own low-cost, tangible, and high-impact educational models.

## Conclusions

The low-cost, high-impact Ball-and-Balloon activity is an innovative and highly effective teaching-learning strategy for abstract concepts in pharmacology. It resulted in a statistically significant increase in student knowledge and promoted a palpable shift toward a more positive, engaged, and motivated attitude toward the subject. By converting the abstract mechanism of drugs acting on the uterus into a tangible, experiential model, this study provides a powerful, replicable, and scalable template for medical educators. This innovation successfully bridges the resource gap, meets the demands of contemporary CBME and AETCOM competencies, and underscores the potential for simple, creative solutions to elevate the quality of medical education in resource-limited settings globally.

## Appendices

Pre-test questionnaire (prevalidated)

1. Oxytocin produces uterine contraction by binding to which type of receptor on myometrial cells? a) β2-Adrenergic receptor b) Ligand-gated Na⁺ channel c) G-protein-coupled oxytocin receptor d) Tyrosine-kinase receptor

2. In late pregnancy, oxytocin mainly contracts which segment of the uterus? a) Cervix b) Lower uterine segment c) Fundus and body d) Whole uterus equally

3. Which oxytocic is CONTRAINDICATED in severe hypertension or pre-eclampsia? a) Oxytocin b) Dinoprostone c) Ergometrine d) Misoprostol

4. The pharmacological agent most commonly used for cervical ripening prior to induction of labour is: a) Oxytocin (i.v.), b) Dinoprostone (vaginal), c) Tranexamic acid, d) Methylergometrine

5. Low-dose oxytocin is preferred over ergometrine for labour induction mainly because it: a) Causes potent vasoconstriction b) Allows phasic contractions with fetal oxygenation, c) Acts orally, d) Has a longer duration of action

6. Oxytocin controls postpartum haemorrhage primarily by: a) Relaxing lower segment, b) Vasodilating uterine vessels, c) Tonic contraction compressing spiral arteries, d) Increasing platelet count

7. Continuous high-dose oxytocin with large volumes of 5 % dextrose infusion may precipitate: a) Hyperkalaemia, b) Water intoxication/hyponatraemia, c) Hyperglycaemia, d) Metabolic alkalosis

8. Which drug acts as a competitive antagonist at oxytocin receptors to delay pre-term labour? a) Nifedipine b) Atosiban c) Ritodrine d) Carboprost

9. A key safety advantage of oxytocin during induction is its short plasma half-life of approximately: a) 45 s, b) 6-12 min, c) 1 h, d) 4 h

10. Which of the following uterotonics is commonly used in combination with oxytocin in severe postpartum hemorrhage? a) Methylergometrine b) Misoprostol c) Dinoprostone d) Tranexamic acid

Post-test questionnaire (prevalidated)

1. A balloon model shows rapid myometrial contraction after Drug X, mediated by inositol-trisphosphate-driven Ca²⁺ release. Drug X is acting on which receptor class? a) β2-Adrenergic receptor b) Ligand-gated Na⁺ channel c) G-protein-coupled oxytocin receptor d) Tyrosine-kinase receptor

2. In the demonstration, only the fundus of the balloon tightened while the outlet remained soft, allowing descent of the ball. Which physiological property does this illustrate? a) Cervical constriction b) Fundal dominance of oxytocin c) Uniform uterine tetany d) Lower-segment spasm

3. A patient with pre-eclampsia develops uterine atony. Which uterotonic should be AVOIDED due to the risk of dangerous BP rise? a) Oxytocin b) Dinoprostone c) Ergometrine d) Misoprostol

4. Prior to induction, an intravaginal insert is placed. Four hours later, the cervix is soft and dilated, with gentle contractions starting. Which drug was most likely used? a) Oxytocin b) Dinoprostone c) Tranexamic acid d) Methylergometrine

5. During the activity, a peer squeezed the balloon so rigidly at the fundus and cervix that the ball could not descend. Which drug effect was exaggerated? a) Oxytocin b) Dinoprostone c) Ergometrine d) Atosiban

6. Oxytocin arrested postpartum haemorrhage in the mannequin model by which mechanism? a) Relaxing myometrium b) Compressing uterine sinusoids via sustained contraction c) Increasing platelet aggregation d) Decreasing maternal blood pressure

7. A mother on high-dose oxytocin becomes drowsy; labs show Na⁺ 118 mmol/L. The likely pathogenesis is: a) Potent natriuresis, b) ADH-like water retention (water intoxication), c) Renal K⁺ loss, d) Hyperglycaemic osmotic shift

8. To halt pre-term labour, you start an IV drug that competes with endogenous oxytocin at its receptor. Identify the agent. a) Nifedipine, b) Atosiban, c) Terbutaline, d) Carboprost

9. Fetal distress occurs during induction; stopping the oxytocin drip allows uterine activity to return to baseline within 10 min. This safety margin is due to oxytocin’s: a) Poor receptor affinity, b) Short plasma half-life permitting rapid titration, c) Renal excretion, d) Inactive metabolites

10. After elective caesarean section, a single IV bolus of a long-acting oxytocin analogue provides prophylaxis against atony for several hours. Which drug is this? a) Desamino-oxytocin, b) Atosiban, c) Carbetocin, d) Methylergometrine
